# The Use and Misuse of Sedatives and Hypnotic Drugs
*A paper given to the Bristol Medico-Chirurgical Society, on 12th December, 1955.


**Published:** 1957-01

**Authors:** R. E. Hemphill

**Affiliations:** Consultant Psychiatric Physician, United Bristol Hospitals and Bristol Mental Hospitals. Lecturer in Charge, Department of Psychiatry, University of Bristol.


### THE USE AND MISUSE OF SEDATIVES AND HYPNOTIC DRUGS*

BY

R. E. HEMPHILL, M.A., M.D., D.P.M.

*Consultant Psychiatric Physician, United Bristol Hospitals and Bristol Mental Hospitals.*
*Lecturer in Charge, Department of Psychiatry, University of Bristol.*

Sedatives have come to occupy a unique place in medical practice. Their an[illegible] consumption in this country now probably exceeds that of all other drugs put toge[illegible] In the U.S.A. in 1948 the production of barbiturates alone amounted to 336 [illegible] or the equivalent of 24 therapeutic doses per inhabitant (W. H. O. Report, 19[illegible] and it is certainly greater today. However, sedative therapy is comparatively mod[illegible] and until the nineteenth century was confined to the alleviation of pain.

In the therapeutics of the ancients, hypnotics and sedatives rarely figure; in[illegible] more attention was paid to somnolence than to insomnia or restlessness, and mas[illegible] diet, purges, exercise and bleeding were the usual treatments for mental and neu[illegible] disorders. Opium and mandrake—ancient remedies—were recommended for [illegible] casional use in the relief of pain. They were probably not used for what would no[illegible] called nervous conditions although something similar to anxiety was known and [illegible] been described under the title of "watchfulness". Paulus Aegineta, in his celeb[illegible] synopsis in the seventh century, distinguishes between watchfulness proceeding f[illegible] pain and fever and the watchfulness of those in health, and of the latter he w[illegible] (1844):

> "If their watchfulness proceeds from sorrow, care or any mental emotion, we [illegible] endeavour, if possible, to remove the offending cause and then to divert the atte[illegible] by agreeable sounds. For this purpose, some seek after the gentle noise of w[illegible] by which they are soothed and lulled to rest. After proper digestion they sh[illegible] use baths, especially in the evening, and a moistening diet. They may also mix [illegible] green leaves of the black poppy with condiments and eat fish of easy diges[illegible] They should also use plenty of wine which is light and not old. I have kown [illegible] succeeding suddenly to fatigue produce the effect. Moderate coition will somet[illegible] do the same."

To seek for a cause of the mental distress first would be good advice in psychiatry [illegible] general practice today.

Sedation by day is a modern introduction. It is not mentioned by the older En[illegible] classical novelists. Charlotte Brontë writing in 1853 describes in *Villette* the u[illegible] sedatives at that date: "The sedative had been administered; in fact they had g[illegible] me a strong opiate—I was to be held quiet for one night." But by the end o[illegible] century bromides and valerian as daily sedatives were recommended in stan[illegible] textbooks of medicine. The term "sedation" is loosely used today and expres[illegible] such as "adequately sedated" are without any real meaning. However, the [illegible] "hypnotic" is precise and of ancient origin being "a medicine that causes sle[illegible] Interest in hypnotics, other than opium, alcohol and cannabis indica, dates from [illegible] discovery of anaesthesia, and the search for anaesthetic substances produced a nu[illegible] of new hypnotic and narcotic drugs such as bromides, chloral hydrate, sulphonal [illegible] paraldehyde. After a considerable vogue in the last century, they have now [illegible] practically superseded by the barbiturates and newer hypnotics. The histor[illegible]

* A paper given to the Bristol Medico-Chirurgical Society, on 12th December, 1955.

modern sedation is the history of the use of the barbiturate drugs and of the successful and lavish pharmaceutical publicity that has accompanied it.

### BARBITURATES: THEIR HISTORY AND PROPERTIES

Barbitone, the oldest barbiturate, was first introduced into medicine by Fischer and von Mering in 1903; sold as "veronal", it is still occasionally used. It was followed in 1912 by phenobarbitone under the trade name "luminal", still prescribed as an anti-convulsant. During the next few years, over 2,000 barbiturates were synthesized and studied, and about 50 were put on the market (Goodman and Gitman, 1955).

The modern barbiturates have the advantage of lighter action and shorter duration than veronal and phenobarbitone. All the barbiturates are capable of producing sleep and are used more for the treatment of insomnia than anything else. With adequate oral dosage, sleep is induced in less than one hour from ingestion. It is said to resemble normal physiological sleep and to be easy, tranquil and dreamless. In spite of these claims, there is often a hangover after awakening without necessarily any physiological disturbance. Barbiturates produce a depression of activity of the central nervous system and are capable of altering the pattern of the electroencephalogram. Any degree of reduction in central nervous system activity can be produced from drowsiness to deep coma. Nevertheless, the effect cannot always be easily predicted. It has been observed that the intensity of the effect depends not only on the particular barbiturate, the route of administration and the dosage, but on the level of reflex excitability of the patient at the time when the drug is administered. The attitude of mind of the patient to the situation, as well as to the drug and the illness, is of importance. This is true even for animal experiments. Chen (1954) has related how, when he prepared a monkey for demonstration of the effect of quinalbarbitone for a congress and had taken the precaution of anaesthetizing it twice previously by way of rehearsal, after an adequate dose of the drug, the monkey sat up restlessly and the demonstration was a failure. Suspicion of the audience had alerted it and overcome the soporific effect.

Sometimes, individuals under stress become more wakeful when damped by barbiturates. Wendt (1954) has reported that a group of volunteers, who had been kept alert for 40 hours, became irritable, anxious, apprehensive and difficult to keep awake; 100 mg. of secobarbitol aroused them sufficiently to continue with further experiments during the night. This paradoxical reaction should be borne in mind in treating the insomnia of patients who have active causes for alertness and anxiety. It also explains why a single dose of the barbiturate may reduce tension and improve the performance of an individual in acute stress. Disadvantages of barbiturates are their effect on depressing the respiratory centre; their tendency to produce dependency and sometimes addiction; and the hangover effect.

Barbiturates are grouped as light, medium and heavy, according to the rapidity of their action and its duration. The heavier barbiturates are slower in action, but the action is more prolonged, and the depressing or damping effect is clinically greater and therefore the tendency to hangover is also greater. They are probably more dangerous instruments for suicide.

Such has been the power of advertising, that the barbiturates in common use are referred to by their proprietary names such as Nembutal, Soneryl, Sodium Amytal, and so forth. The use of trade and chemical names is most confusing, and the confusion is made still deeper by the various coloured capsules in which the drugs are usually supplied. The names and the colours lead to nicknames, such as "red devils" or "yellowjacks", which have the effect of popularizing the drugs and fixing them in the memory of the public. Patients have been known to say that one doctor used yellow capsules for ease of dispensing because his partner used blue. White pills are now commonplace and many patients do not really trust white capsules any more. With so many proprietary preparations available, it is wise to stick to two or three barbiturates. There has recently been a tendency to market barbiturates in mixtures on the assumption that a combination of a light barbiturate with a heavy one, or both w some other substance, packed in special capsules with different rates of absorpti produces a slow, steady absorption of the sedative over a long period of time thereby tranquillizes the patient. It has yet to be shown that these mixtures have advantage over the single and probably cheaper preparations.

Barbiturates alone have no analgesic properties and may even excite or intoxic patients suffering physical pain. It is claimed that the addition of barbiturate aspirin enhances the analgesic effect and mixtures of this sort may be superior codeine in the relief of mild pain such as toothache or slight injuries.

Some of the more commonly used barbiturates with their trade names and doses

| | | |
|---|---|---|
| Quinalbarbitone (light) | Seconal | 3 gr. (0·2 g.) |
| Amylobarbitone (medium) | Sodium amytal | 3 gr. (0·2 g.) |
| Butobarbitone (medium) | Soneryl | 3 gr. (0·2 g.) |
| Pentobarbitone (light) | Nembutal | 3 gr. (0·2 g.) |
| Phenobarbitone (heavy) | Luminal | ½–2 gr. (32 mg.–120 mg.) |
| Barbitone sodium (heavy) | Medinal | 5–10 gr. (0·3 g.–0·6 g.) |

### NON-BARBITURATE HYPNOTICS

A number of hypnotics have been introduced as an alternative to barbitura Methylpentynol or "Oblivon" is probably the best known. Its hypnotic potency is high and it is claimed to be non-addiction-forming, produce no hangover and little toxic. In spite of the claims, some deaths attributed to over-dosage have b recorded and Glatt (1955) has described certain alcoholics who had become addic to Oblivon, either in addition to or as an alternative to alcohol. It is of special portance for it can be obtained, as Schedule I, without medical prescription, and some reputation because of its honorary title of "the confidence drug". Methylpent seems undoubtedly to reduce apprehension and give an illusion of contentment calm, even amounting to elation. Candidates, after a successful interview or a s cessful viva, have attributed the result to the drug; and a series of volunteers exam by a police surgeon Sinton (1954) with the "drunk in charge" tests did better normal. Hare (1955*a*) found with patients in Dundry Villas Neurosis Centre *self-controlled* trial that methylpentynol was inferior to Carbromal, which in turn inferior to butobarbitone for securing sleep. It undoubtedly is of value and prob has a special advantage in the treatment of insomnia in old people who are o susceptible to barbiturate and bromide intoxication. Carbromal ureide, simila Adalin and Dormiprin, has about the same hypnotic strength as methylpent dose 12 gr. Generally regarded as harmless, it may cause dermatitis and as it ca obtained without medical prescription cases of addiction or at least drug depend occur. Persomnia resembles carbromal; and carbrital, which is in fact carbromal pentobarbitone, has some vogue.

### SLEEP

Insomnia—the inability to sleep normally or to sleep at all—is feared by all kind people. Throughout the ages, writers and poets have extolled the gift of deep nat sleep that refreshes the body and shuts out care. These imaginative writers dread dreams that spring from the active unconscious, in contrast to those of children of pleasant phantasies and wish-fulfilments.

Keats speaks of "sleep, the soft embalmer of the still midnight"; and he giv clinical indication for a hypnotic drug for anxiety and insomnia when he writes:

> "Save me from curious conscience that still lords
> Its strength o'er darkness, burrowing like a mole.
> Turn the key softly in the veiled wards
> And seal the hushed casket of my soul."

A good hypnotic should be certain in action and produce sleep from which patient awakes refreshed, without hangover. Although hypnotics cannot dire ttack the cause of sleeplessness merely by securing a good night's rest, they may—y interrupting a succession of sleepless nights—make the patient better able to cope vith the mental or physical circumstances that have been initially responsible for the nsomnia.

The aim in the treatment of insomnia should be to secure a reasonable amount of leep for the patient. It is important to inquire what have been the usual sleeping abits—the hour he normally went to bed, how long he slept, the usual depth of sleep, vhether a light sleeper or not. Since most people sleep for many more hours than is ecessary to repair their fatigue, it is not to be expected that a long sound night's sleep an be obtained if the patient is troubled by real worries or anxiety disturbances in the nconscious, without heavy doses of a hypnotic.

### TYPES OF INSOMNIA AND THEIR TREATMENT

The causes of insomnia can be grouped as:

(*a*) Over-excitement and anticipation.
(*b*) Anxiety and psychoneurosis.
(*c*) Psychotic conditions, such as mania or depression.
(*d*) Disturbed sleep pattern in senility.
(*e*) The loss of the sleep habit.
(*f*) Pain and physical illness.

(*a*) *In over-excitement and anticipation* (for example, before a wedding or an examiation) the subject may get no sleep at all without a hypnotic. If a hangover is to be voided, he should not be given the drug late—probably not after midnight. If ossible he should go to rest at about 10, then when all is quiet take Seconal gr. 1½ or 3, r Oblivon 0·5–1·0 g., or corresponding drug. However, it commonly happens that e goes to bed late, lies awake until the small hours, then in desperation takes a drug, ets some broken sleep, and has an unhappy hangover in the morning. A quickcting barbiturate is best and should be given in larger rather than smaller dosage rovided it is taken not less than 8 hours before rising.

(*b*) *Anxiety and psychoneurosis* are by far the commonest and most important causes f chronic insomnia. Acutely anxious and worried patients dread the night with its ension, restlessness and nightmares. Preoccupied with their worries or fears and erhaps rendered inefficient by daily sedation also, they find the day intolerable. They hould not be permitted to go to bed too early as they are eager to do so to shorten the ay, and as they desire a heavy dreamless sleep, they rapidly become dependent upon rugs. It will be observed that they become tense and restless towards evening, impatient for the drug. If it is given early the dose will have to be increased considerably lereafter. Eventually the patient may demand ten hours' sleep at the cost of a heavy angover in the morning.

It is important to distinguish between the hangover effect and morning discomfort ue to anxiety. The characteristics of hangover are weary, dull awakening, headache, thargy and indigestion. The patient commonly says he "feels awful", but improves owly as the day goes on, and he attributes this to his illness. As the hangover effect ears off, the patient becomes livelier, but towards evening he becomes tense and alert anticipation of the night. The typically anxious patient awakes tense, alert, with astro-intestinal uneasiness, palpitations, vasomotor upsets and a sense of panic. This is probably due to the disturbing effects of fears from the unconscious which have een operating during sleep. As the day goes on and he has more to do and more outlet om his tension, these fears sink back and he feels calmer towards evening.

The hangover can be responsible for inefficiency and inability to cope with work or ouse which may have more serious consequences than the original circumstances. It this fact, probably more than anything else, that makes a neurosis chronic. These condary problems produce more anxiety with an increasing demand for drugs and a cious circle is easily established.

Hypnotics are important in the treatment of acute situations, acute anxieties, bere[illegible] ments and worries. In order to avoid habit formation, it should be made clear to [illegible] patient that the securing of sleep, desirable as it is, is not in itself a cure, and that [illegible] if the patient is unable to sleep without drugs after having solved some of the difficu[illegible] of the anxiety situation, he cannot continue to have nightly hypnotics. It is q[illegible] true that the publicity literature suggests that barbiturate sedation can be contin[illegible] year after year, keeping the machine of the mind or the brain in a sort of bala[illegible] state. This is not so, and the need for hypnotics generally springs from ner[illegible] illness or real worries, which will have to be solved.

In insomnia due to recent events it is wise to prescribe nightly drugs for a wee[illegible] two, but in long-standing cases the procedure is different. After a patient has had [illegible] or three nights' sound sleep he should be instructed to do without drugs for the t[illegible] or fourth night if possible, even at the expense of a restless night, and should be [illegible] that it is important not to take sleeping pills on more than three nights in a week th[illegible] after. Many patients believe that sleeping pills are harmless and are not what they [illegible] "drugs". They would treat them with greater respect if they knew they were po[illegible] and dangerous. The capsules should be given into the charge of a husband or wif[illegible] other relative, who should be instructed to keep a check on the amount consum[illegible] this is often much greater than anyone suspects. Determined patients may persu[illegible] their friends or servants to give them drugs provided by their own doctors for all[illegible] "nerves", or to obtain prescriptions from private practitioners as well as from [illegible] own National Health Service doctor.

(*c*) *Psychotic conditions*, of which mania and depressions are the commonest. *Mania* is unlikely to be treated for long outside hospital. Patients are consist[illegible] restless and not only do they not desire to sleep but they can do with very much [illegible] sleep than normal individuals. Paraldehyde *dr.* 2 or 4 repeated during the nigh[illegible] medinal gr. 10 or gr. 7 plus paraldehyde dr, 2, will probably secure a night's rest[illegible]

*Depression.* One of the characteristics of depression is inadequate sleep by [illegible] with early awakening followed by mental and physical retardation and inactivity by [illegible] Sedation by day is likely to deprive the patient of any chance of sleeping at nigh[illegible] should be avoided. Sedation at night, preferably, by means of Sodium Amy[illegible] Nembutal or Paraldehyde, should be given at a time when the household is going to [illegible] It is essential that the depressed patient should not be out of observation. The pra[illegible] of giving a sedative drug to a depressed patient early, while the rest of the famil[illegible] downstairs to watch television is an invitation to suicide. Also because of the [illegible] awakening, late administration is of greater value.

(*d*) *Disturbed sleep pattern in senility.* The restlessness of the senile is a con[illegible] problem. Here there is much to be lost and nothing to be gained by permittin[illegible] patient disturbed nights. These patients, by reason of brain-wasting and poor cer[illegible] circulation, are particularly troublesome at night when brain metabolism is low. [illegible] become delirious, get up, and may wander from the house and come to harm. [illegible] probably do not require much sleep at night as they doze off frequently by [illegible] Paraldehyde, dr. 2 or 4, when the household is going to rest or when the pati[illegible] getting troublesome, is by far the best drug. As an alternative, Oblivon 0·5–2 [illegible] can be used, but barbiturates usually excite and the bromides rapidly produce a [illegible] confusion. An increase in aimless restlessness with deepening confusion should [illegible] suspicions of bromide or barbiturate intoxication which is confirmed if heavier [illegible] fail to calm the patient. Chlorpromazine (Largactil) has definitely some advan[illegible] in the treatment of old patients, and in 25 mg. doses t.d.s. will often secure a [illegible] reduction in their aimless agitation (Seager, 1955).

Most important in the care of the sleepless, restless senile is to establish a re[illegible] routine—an exact hour for going to bed, and for getting up, for attention to bo[illegible] and washing, for meals and recreation.

(*e*) *The loss of the sleep habit* may occur in anxious women who have been kept a[illegible]

at night by a baby or who have kept alert in nursing a sick child, or in nurses and others who work night shifts. The habit of sleep is lost and a faulty habit established. These cases are most difficult to deal with. The trouble usually occurs in tense, worrying individuals, normally light sleepers. Irrespective of their work, routine, or amusements, they cannot get to sleep, they fear the hours of the night and at the best doze fitfully. They become intolerant of the noises of the night, the child's cough or the husband's breathing, the ticking of the clock for example and they are genuinely fatigued during the day. The treatment aims at breaking the chain of sleeplessness by hypnotics and then establishing a normal routine. Since these weary individuals usually go to bed early, they should go to bed late and be given a hypnotic in an adequate dose for about 7 hours' sleep, for three nights running. Thereafter they should try to do without the hypnotic, but if sleep still does not come, take a dose late on occasional nights. A visit to the cinema, a dance or some other recreation in the evening is a great help.

(*f*) *Pain and physical illness.* The management of insomnia due to physical disturbances is, of course, bound up with the treatment of the physical condition.

### SEDATION AND SEDATIVE DRUGS

Sedation is the attempt by means of drugs to secure mental and physical tranquillity in an individual who is tense, restless, strung-up or agitated. These expressions of subjective feelings indicate a good deal of sympathetic disturbance, and they also indicate that the patient is receiving impulses of a disagreeable character from inside. Barbiturates will not directly reduce tension, but small doses may calm the overwrought patient; they also make him less receptive to stimuli such as noises, children crying, that provoke attacks of nerves, anger or panic, and to the stress of situations. In large doses they are likely to cause mental dulling and hangover feelings that add considerably to the feeling of physical illness. The medium and heavy barbiturates should be used, and small doses of phenobarbitone or Sodium Amytal are often of great benefit, especially at the time of acute stress. A small dose on rising often helps to relieve the tension and turmoil of an acutely anxious patient and enables him to start the day's work with some confidence. Sedatives should not be taken regularly, if possible not more than once daily and they should not cause drowsiness. Regular barbiturates by day do more harm than good.

In very acute, disturbed, anxious cases, barbiturates for a few days will certainly ease the patient and blot out outside worries, and in severe panic or stress a single full hypnotic dose of barbiturate to secure sleep is often necessary. Some people are helped by a small dose of phenobarbitone of Sodium Amytal before interview or a stressful event. This is, of course, because it makes the individual less alert and less receptive, so that the situation does not produce the reflex disturbances—palpitations, dizziness and gastric upset—that it otherwise would.

There is not much evidence that bromide mixtures have a sedative effect, and as bromides are cumulative and intoxication is so easily produced, it is probably wiser not to prescribe them.

Tension in the head or the muscles, with a boiling-up feeling inside, is one of the commonest and most distressing complaints of psychoneurotic patients. There is good reason to believe that these feelings are due to an increase in muscle tone and one has only to look at over-wrought patients to see how strained and tense they are. For example, a highly successful business man from his early days would press his knees against his desk so fiercely when putting through important deals that he wore out his trousers—not a great expense when a quarter of a million was involved. For the last twenty years his desks have had a recess cut out. But apart from such obvious reactions many patients are never free from a feeling of aching tightness in their muscles, particularly involving the head and back of the neck. This has led to the search for drugs that might reduce muscular tension by operating on the musculature or the autonomic nervous system. Berger (1946) and Stephen (1947) demonstrated t Mephenesin could produce muscular relaxation accompanied in human subjects b feeling of euphoria. However, the reports of different surveys of the use of Mephene in anxiety and tension study have been conflicting and on the whole not encourag

A study with female neurosis patients, carefully conducted by Hare (1955b) Dundry Villas, Barrow Hospital, showed no greater improvement with Mephene than with a placebo, and Mephenesin was responsible for sensations of a disagree character not necessarily connected with relief of symptoms. Preparations of Mephe sin with a combined barbiturate do not seem to have any advantage over the sim barbiturates.

Combinations of amphetamine with barbiturate of which "Drinamyl" is an exam have had a considerable vogue. This is a curious example of paradoxical reasoni the supposition being that the barbiturate offsets the tension-producing qualities of amphetamine, which in turn reduces the depressing effect of the former. It is doubtful whether these preparations have any special indications or value.

*Chlorpromazine* (*Largactil*). This drug, about which an enormous amount has b written, partakes of the nature of the antihistamines. Many claims have been made it, but it is true to say that there is no agreement about its real use or indications psychiatry. In some cases of chronic psychosis, chronic schizophrenia, mania, sta of agitation, it has produced quite remarkable results. While so far there are no me of predicting in which cases it will be of value, it has revolutionized social life in large wards of chronic and disturbed patients in some hospitals. As a result it has a publicity out of all proportion to its usefulness and has been referred to as "chemi leucotomy" in the non-medical press. Even in psychotic cases in which there maximum benefit the effect is usually not long maintained after the drug has be withheld. Because of its toxic effects it should not be administered for more than fo weeks unless there has been considerable objective improvement. Chlorpromaz has little immediate sedative effect and should not be used as an alternative to ot sedatives for acute excitement, acute agitation and acute anxiety attacks. Many co sider it is not suitable for out-patient use. In order to secure an effect it must be gi in fairly heroic doses, about 25 to 75 mg. t.d.s. and some workers consider that on first three days it should be given intramuscularly. The main danger is hypotens attacks, which is very real with the intramuscular route, and if this is used patie should be kept in bed lying flat during this period of treatment. In small doses it no sedating effect and produces often an upset stomach, a dry mouth and a cert amount of general malaise. Most workers have seen cases in which it produced jaund There is some evidence that in very tense, agitated individuals, combined with b biturates, it does produce a considerable degree of alleviation of agitation. As a gene rule its use should be confined to in-patients, with the exception of the treatment of aged.

*Rauwolfia serpentina*. This is one of life's swindles. It has been widely publici as a sedative or nerve tranquillizer. Until recently it could be obtained without f mality from the ordinary chemist. Advertisements for preparations containing rauw fia appear in the daily papers. The typical example in a Bristol daily paper in Octo 1955 urged a form of treatment to secure serenity by day without sleepiness, untroub rest by night. The new mode of calmness and quiet confidence then produced wo be maintained thereafter by taking two tablets daily. In the advertisement rauwo was credited with being widely prescribed for its effectiveness for relieving tensi and nervousness and the preparation advertised containing rauwolfia was stated be harmless and non-habit-forming.

Some firms have now agreed not to supply it without prescription, but the publici remains. It is capable of producing a drop in blood-pressure even in normotens individuals. There is no satisfactory evidence that any good has come from its use psychiatry. Leitch, Hare and Seager, Barrow Hospital (1956) have compared effect on neurotic anxiety with that of Sodium Amytal in a well-controlled grou

Most patients reported some alleviation of symptoms with Sodium Amytal, but there was no tendency to alleviation with reserpine. This is a particularly important finding, because with the patients selected and the conditions under which the test was made, one might have expected some improvement with almost any drug, and to a greater extent than with unselected patients treated outside.

Reserpine has definite disadvantages and many cases have developed severe psychotic depression as a result of treatment with it for hypertension. This depression does not necessarily clear when the drug is withdrawn and may be due to organic brain change produced by the reserpine directly or indirectly. One of the great pharmaceutical firms has already issued warnings about the danger of depression. At the present time it is only to be condemned in the treatment of psychiatric conditions.

*Acute psychiatric emergencies.* A practitioner is sometimes called upon to deal with wild excitement in an alcoholic, acute mania, or some acute disturbance—perhaps due to G.P.I. or delirium tremens. The best emergency treatment still is injection of hyoscine and morphia, with or without atropine; 1/75 hyoscine and ½ gr. of morphia may suffice; and 1/50 hyoscine with ½ gr. morphia will quieten most men and enable necessary action to be taken in peace. Barbiturates and mixtures such as somnifaine are ineffective and more toxic. Intramuscular paraldehyde is probably not very satisfactory and there is a risk of injection abscess.

*Toxic complications by sedation.* Confusion with restlessness, which is not improved by barbiturates, is often due to drug intoxication. This is particularly true for barbiturates or bromides in patients who are under-nourished or physically ill. In drug intoxication an increase in restless confusion and delirium will follow the giving of more drugs, for the hypnotics—by interfering with the metabolism of the brain—now act as an exciting agency and not a tranquillizer. The drugs should immediately be stopped and if possible the patient got into hospital. Paraldehyde and morphia are probably safe in these cases and do not seem to accentuate the confusion, but it is preferable to withhold all hypnotics if possible for a couple of days, in order to give the brain a chance of recovering. In confused seniles, heavy dosage with Vit. B group sometimes reduces the confusion dramatically. The dangerous consequences of taking barbiturates and alcohol together are well known even by alcoholics. The nervous public and after-dinner speaker should beware of taking the dose of barbiturate referred to before if he is likely to drink much alcohol before speaking otherwise he may find his speech ataxic and his ideas incoherent.

### PSYCHOGENIC OR PSYCHONEUROTIC PAIN

The test of a good analgesic is its ability to relieve the patient of pain and a test for organic pain is whether it can be relieved by analgesics. Many psychiatric patients complain of low-grade pain for which no organic cause can be found. It will be noticed that when attention is distracted the pain seems to disappear. There patients get little or no relief with analgesics such as aspirin, and this fact is a good point of differential diagnosis between organic and non-organic pain.

Analgesics reduce the appreciation of pain so that it makes less impact on patients' consciousness. Psychoneurotic pain is probably produced by the increased awareness the patient has of his body and the extent to which sensations that normally would not amount to more than discomfort attract his attention. Analgesics will not alter this attitude of mind and will not improve him. This is particularly true of psychoneurotic headaches or headache in compensation cases after an old concussion. Relief with analgesics is very rarely found. These patients should not be treated with sedative and narcotic drugs, as these conditions are very intractable. Once the patient has started to take drugs he may be unable to give them up. Barbiturates often seem to increase discomfort and give more substance for the elaboration of pain. Similarly it is seldom wise to give barbiturates or codeine to patients between attacks of migraine. The result often seems to be a more or less continuous headache and dulling of cerebration.

### THE PATIENT'S POINT OF VIEW

What is the patient's attitude to sedation and his point of view? It is often surpris sensible. Very often demands for sedation are not so much due to the desire o patient to dictate his treatment as to his belief in the value of sedation and in the dom of his doctor. Patients always discuss their symptoms in waiting-rooms, patient clinics and especially in the neurosis centre. When they learn what has prescribed for others they expect it for themselves. Patients will rightly argue if their doctor did not believe in the medicine he prescribed he would not pres it for them. Sedatives are bound to dull cerebration, so if the patient does not im soon he will suffer from dullness and imagine this to be a result of or a deterior in his illness. It would be very helpful if after a week or two the patient was asked thinks the capsules have brought relief or if there have been any ill-effects or symptoms; in the latter they should be stopped or changed and the patient tol reason why.

Patients believe nowadays that there are nerve tonics which will help them to bat anxiety or nervous troubles and the battles of life, and they often imagine barbiturates or bromide mixtures are nerve tonics. For these credulous patie harmless bitter or glycerophosphates, prescribed for a few weeks, may be very valu for this is preferable to using depressing drugs, provided its real nature is not discl

The effect of replacing sedatives by an inert medicine may be dramatic. Pat often complain that they cannot take any more of the tonic—it was too strong—i made them too lively, and so after two or three doses they took none at all, to its effects to settle down. Naturally this is a very hopeful sign, as long as one doe explain to the patient that he is merely experiencing relief from sedation and the of improved health and alertness. It is wiser to keep the explanation secret, for i event of a relapse, which often happens, one does not get a second chance.

To use the word "placebo" is a bit unfair. It is inherent in human nature i cepting a National Health Service to expect that medicines exist that will alle common bodily and neurotic disabilities, and to deny a patient a prescription often impairs his confidence. On the other hand, a simple prescription for a period of time gives him positive suggestion and valuable support. It is very rar a patient to come to the conclusion by himself that medicine is making him w

### SUICIDE AND ACCIDENT

The numbers of suicides and accidental deaths due to taking hypnotics and narc is enormous and by far the greatest number of cases are due to the barbiturates. average annual number of suicidal and accidental deaths due to barbiturates in period 1950–53 was more than ten times that for the period 1931–35. Figures supp by the *Registrar-General's Statistical Review of England and Wales* (1952) (for adults 15) were: 396 cases of accidental poisoning, of which **212** were due to hypnotics analgesics and 184 to all other kinds of chemicals. In the same year there were suicides due to poisoning in England and Wales of which **458** were due to hypn and analgesics and 263 to other substances.

For 1953 the figures are much the same. There were 281 accidental poisonin **188** due to narcotics and hypnotics, and 93 to all other chemicals. There were suicides, of which **447** were due to hypnotics and 180 to all other poisons.

These figures are obviously very incomplete and the numbers of cases of attem suicide by barbiturate poisoning in the country are many times greater. Possibly proportion of attempted to successful suicides by taking drugs is 10 or 15 to 1.

In 1954, in one of the Bristol hospitals, 57 cases of attempted suicide were admi 33 were attempted by soporific substances and 24 by all other methods including and self-inflicted injuries. There were 2 deaths in each group. Eight cases of accid poisoning were admitted, of which 6 were due to barbiturates, 2 to aspirin and cylates; there was one barbiturate death.

When it is realized that nearly all the hypnotic poisons were obtained on medical escriptions and became available in this way and this way only, and that the quanti-s necessary to cause each death were large, it makes one reflect on the seriousness of escribing barbiturates lightly and in large amounts.

Capital punishment and murder are very much in the news. It is reckoned that ere are about 140 murders per year and perhaps a dozen murderers are hanged. hus the activities of murderers and of justice together account for only about 150 es per year—sedatives for over four times that number, and there seems to be no ubt that the number of suicidal attempts with drugs is increasing. Although, as a ding article in the *Lancet* (1954) said, there is a cloud over the barbiturates, there ems to be no real alternative to them as yet.

Dispensing in capsules allows a patient to carry a lethal dose about with her con-niently, and many do, in their bags or pockets. The number of accidental deaths, ne of them children and suicides, show that keeping dangerous drugs in antity about the house is exceedingly risky. The risk is much reduced when liquid edicines are issued in bottles. The risk of over-dosage, such as taking four capsules stead of one, is also less. As long as patients are allowed to carry barbiturate capsules osely in their pockets, they will not respect them or fear them. In an attempt to duce the risk of suicide capsules of barbiturates have been packed with a small antity of an emetic or picro-toxin to act as an emetic or antidote in the event of over-sage—so far this promising idea has not proved very practicable for various tech-cal reasons (*B.M.J.* 1954).

### SOME CONCLUSIONS

Causes of the modern vogue for sedatives and hypnotics are complex. The increased sh of life and the orientation of medical thought are only partly responsible. The eat general public, as the manufacturing chemists know so well, is a potential patient, d advertising publicity sees to it that the public is reminded from the cradle to the ave of its weak nerves and that sleep is essential to health, but is hard to get. Simple mulants, like tea, are no longer sufficient. Aspirin, after a long run, and after the ief flirtation with science that produced advertising diagrams of the brain and of the ctroencephalogram, has been humbled to the grocer's shop.

Food producers claim their share. In full view of the Bristol Royal Infirmary is a mmand to "take milk for weak nerves", and we can hardly hope to escape seeing in ery city today a model balancing a glass of milk on her finger in bed advertising milk r sound sleep. The brewers' campaign with their stouts and "night starvation" has en added to the terrors of the night. But when fashions have changed and advertising pushing products for keeping the public awake, there will still be the hard core of ug-taking patients, the despair of general practitioners and psychiatrists alike, whose mplaint was so well and critically expressed by a patient recently: "You're a doctor medicine, aren't you, well then why don't you give me some?"

### *BIBLIOGRAPHY*

Berger, F. M., and Bradley, W. (1946). *Brit. J. Pharmac.*, **1**, 265.

*B.M.J.* (1954). **2**, 294.

Chen, K. K. (1954). *Symposium on Sedative and Hypnotic Drugs.* The Williams and Wilkins ., Baltimore, 54.

Glatt, M. M. (1955). *Lancet*, **1**, 308.

Goodman, L. S., and Gilman, A. (1955). *The Pharmac. Basis of Therapeutics*, 2nd Ed. The acmillan Co., New York. 123.

Hare, E. H. (1955*a*). *Brit. J. Prev. and Soc. Med.*, **9**, 140.

Hare, E. H. (1955*b*). *J. Ment. Sci.*, **101**, 172.

*Lancet* (1954). **2**, 75.

Leitch, A., Hare, E. H., and Seager, C. P. (1956). *Lancet*, **1**, 545.

*Registrar-General's Statistical Review for England and Wales*, 1952. H.M.S.O. 1953. *Ibid.*

Seager, C. P. (1955). *B.M.J.*, **1**, 882.
Sinton, A. S. R. (1954). *Lancet*, **2**, 54.
Stephen, C. P., and Chandy, J. (1947). *Canad. Med. Assoc. J.*, **57**, 403.
*The Seven Books of Paulus Aegineta.* Translated from the Greek by Francis Adams. Syd[illegible] Soc. (1844). Bk. I, 181.
Wendt, G. R. (1954). *Symposium on Sedative and Hypnotic Drugs.* The Williams and W[illegible] Co., Baltimore, 101.
*World Hlth. Org. Techn. Rep. Ser.* (1950). **21**, 13.

